# Late Recurrence of High-Grade Vulvar Leiomyosarcoma After 5 Years: A Rare Case Report and Expanded Review of Reported Cases

**DOI:** 10.3390/jcm14176032

**Published:** 2025-08-26

**Authors:** Angel Yordanov, Ivan Ivanov, Stoyan Kostov, Ihsan Hasan, Vasilena Dimitrova

**Affiliations:** 1 Department of Gynaecological Oncology, Medical University Pleven, 5800 Pleven, Bulgaria; 2 Department of General and Clinical Pathology, University Hospital “Dr. Georgi Stranski”, 5800 Pleven, Bulgaria; posledenzalez@gmail.com; 3 Department of Gynaecology, St. Anna University Hospital, 9002 Varna, Bulgaria; drstoqn.kostov@gmail.com; 4 Research Institute, Medical University Pleven, 5800 Pleven, Bulgaria; 5 Department of Obstetrics and Gynecology, University Hospital “Sofiamed”, 1000 Sofia, Bulgaria; ihsan_hasanov@abv.bg; 6 Faculty of Medicine, Medical University Pleven, 5800 Pleven, Bulgaria

**Keywords:** vulvar leiomyosarcoma, recurrence, diagnosis, treatment

## Abstract

**Background:** Vulvar leiomyosarcoma (VLMS) is a rare and aggressive soft tissue malignancy arising from smooth muscle cells, comprising less than 3% of vulvar cancers. Its clinical resemblance to benign vulvar lesions often leads to delayed diagnosis. Despite surgical resection and adjuvant therapy, VLMS is associated with high recurrence rates and a poor prognosis, and due to its rarity, there is no standardized management or surveillance protocol. **Case Report:** We present a case of high-grade VLMS in a postmenopausal woman, initially diagnosed in 2020 and managed with surgical excision and adjuvant radiotherapy. The primary tumor was a 10 cm solid, lobulated mass involving the mons pubis, with histology confirming high-grade leiomyosarcoma based on marked cellular atypia, high mitotic activity, and smooth muscle differentiation. Immunohistochemistry was positive for SMA, vimentin, and CD34, and negative for S100 and MyoD1. Five years later, the patient developed a local recurrence with an enlarged inguinal lymph node. She underwent complete tumor resection and bilateral inguinal lymphadenectomy. Histology of the recurrent lesion mirrored the initial findings, with no lymph node metastases. This case highlights the aggressive nature and potential for late recurrence in vulvar leiomyosarcoma, underscoring the importance of long-term surveillance. **Conclusions:** High-grade VLMS is a rare malignancy with a high recurrence risk. This case highlights the importance of early diagnosis, radical surgical treatment, and long-term surveillance. Although recurrence occurred five years after the initial treatment, timely surgical intervention led to a favorable postoperative course. Multidisciplinary management and individualized follow-up strategies remain key to improving outcomes in these rare gynecologic sarcomas.

## 1. Introduction

Vulvar leiomyosarcoma (VLMS) is an exceptionally rare and aggressive malignancy derived from smooth muscle cells, accounting for less than 3% of vulvar cancers and an even smaller proportion of all gynecologic malignancies [1]. While the majority of vulvar tumors are benign (e.g., Bartholin gland cysts, fibromas, or lipomas), LMS can clinically mimic benign lesions due to its slow, painless growth in the early stages, resulting in frequent delays in diagnosis and treatment. Histologically, it is characterized by spindle-shaped cells with eosinophilic cytoplasm and pleomorphic nuclei, arranged in fascicles, often demonstrating high mitotic activity and areas of necrosis [1].

Despite complete surgical excision and adjuvant therapy, vulvar LMS is notorious for local recurrence, potential for late metastasis, and poor overall prognosis [2]. The rarity of the disease results in a lack of standardized treatment protocols and limited consensus on optimal surveillance strategies [2]. Therefore, each reported case adds meaningful data to the sparse literature, helping shape future clinical decision-making.

This case report describes a rare instance of recurrent high-grade VLMS occurring five years after the initial curative surgery and adjuvant radiotherapy in 2020 in a postmenopausal woman, as reported by Yordanov et al. [3]. It analyzes the diagnostic challenges, histopathologic features, and immunohistochemical profile of both the primary and recurrent tumor, evaluating any phenotypic changes over time. Furthermore, it presents a comprehensive review of published VLMS cases from the past four decades, examining recurrence patterns, treatment approaches and prognostic factors, with the aim of offering practical recommendations for clinical management and long-term follow-up.

## 2. Case Report

**Figure 1:**
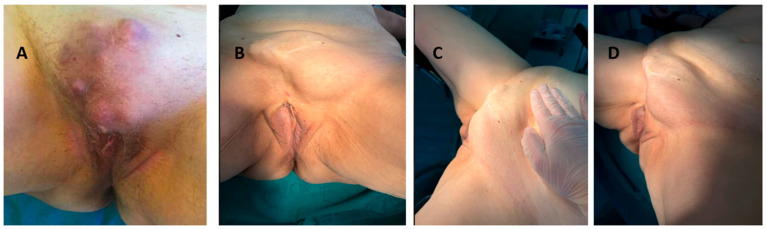
Macroscopic view of the tumor: (**A**) 2020; (**B**) 2025; (**C**) 2025; (**D**) 2025.

Initial diagnosis and treatment in 2020, a 74-year-old postmenopausal white woman, with no significant comorbidities other than arterial hypertension and a prior cholecystectomy (2006), presented with a painful, enlarging mass of the mons pubis, measuring 10 cm in diameter (Figure 1A) [3]. She reported severe pain, partially relieved by analgesics, but denied vaginal bleeding or discharge [3].

Upon examination, a lobulated, mobile, subcutaneous tumor was noted, infiltrating the overlying skin, but not fixed to deep structures. The rest of the gynecological exam was unremarkable.

She underwent wide local excision. Intraoperatively, a solid, lobulated tumor measuring approximately 10 × 10 cm was excised from the symphysis with macroscopically and microscopically clear margins. The wound was repaired in two layers, and the patient had an uncomplicated recovery.

Postoperative recovery was uneventful and the patient began adjuvant radiotherapy five weeks after surgery. Following surgery, the patient received adjuvant radiotherapy consisting of two courses of deep X-ray therapy. The first course delivered 30 Gy as five fractions of 400 R, and the second course delivered 20 Gy as four fractions of 400 R. She completed radiotherapy without complications and remained clinically disease-free for five years.

Five years post-surgery, the patient presented with a recurrent tumor in the mons pubis area, accompanied by an enlarged left inguinal lymph node. A clinical exam confirmed a solid, lobulated formation measuring ~15 × 10 cm and a palpable left inguinal mass (Figure 1B–D). Preoperative ultrasound, Doppler ultrasound, and PET-CT were performed.

**Figure 2:**
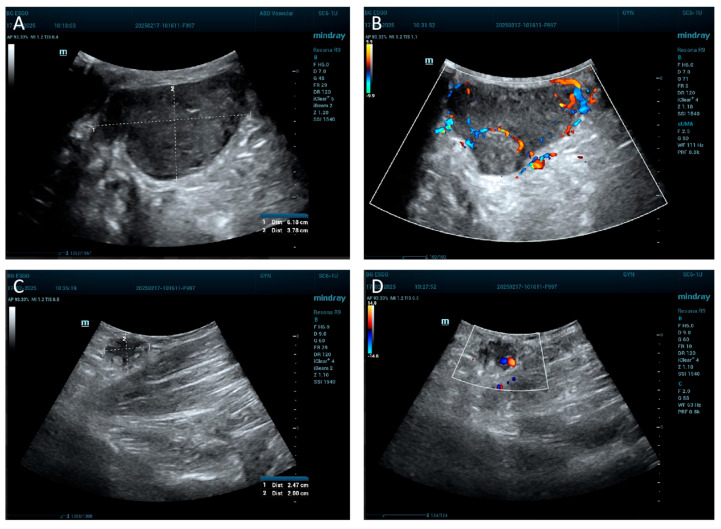
Ultrasound of the tumor formation and lymph node. (**A**) Ultrasound of the tumor formation. (**B**) Doppler ultrasound of the tumor formation. (**C**) Ultrasound of the lymph node. (**D**) Doppler ultrasound of the lymph node.

The ultrasound image shows a well-defined structure with dimensions of approximately 6.18 cm in length and 3.78 cm in width. It appears as a relatively hypoechoic area, darker than the surrounding tissue (Figure 2A). The lymph node ultrasound image reveals a small, well-defined structure measuring approximately 2.47 cm in length and 2.00 cm in width, consistent with an enlarged lymph node. The node is relatively hypoechoic and oval in shape, suggesting a reactive or possibly pathological nature (Figure 2C). A Doppler ultrasound was also performed to further assess vascular characteristics of the tumor formation (Figure 2B) and the lymph node (Figure 2D).

**Figure 3:**
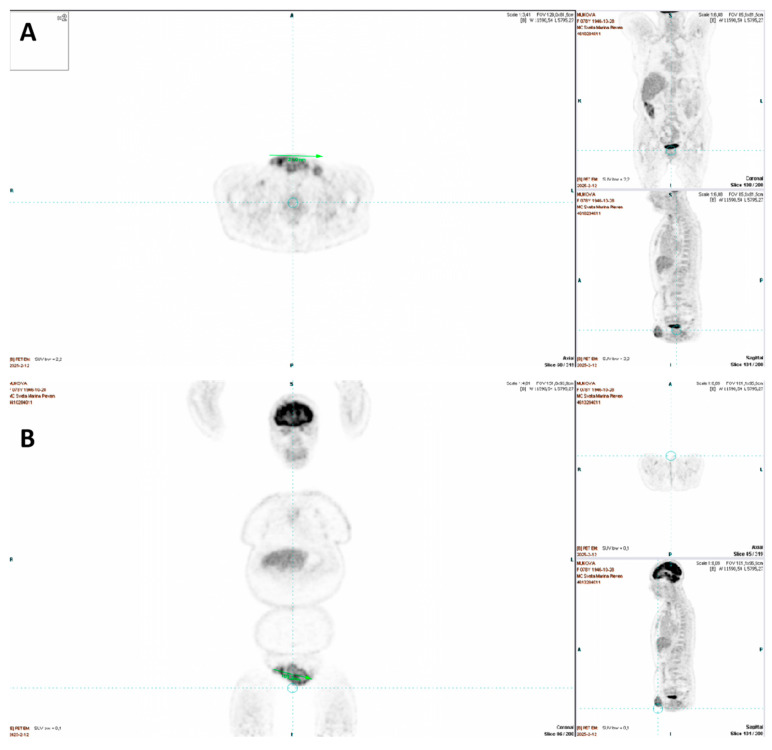
(**A**) PET-CT axial section. (**B**) PET-CT coronal section.

PET-CT shows increased uptake in the symphysis and left pelvic regions, consistent with pathological lymphadenopathy, likely involving the inguinal lymph nodes. No other abnormal metabolic activity is noted (Figure 3).

**Figure 4:**
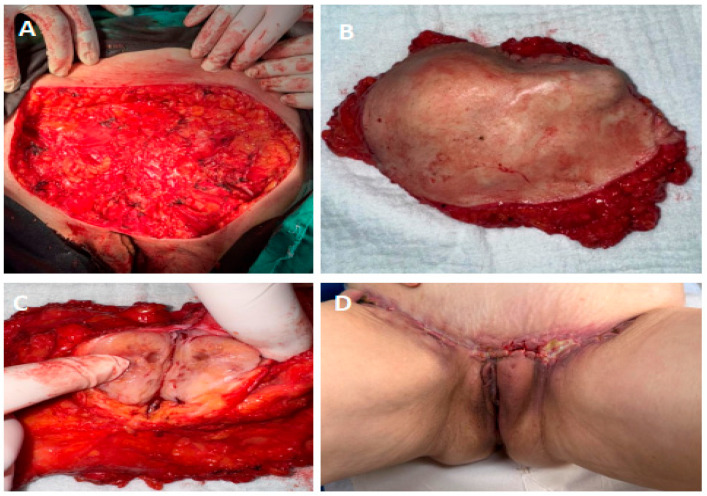
(**A**). Intraoperative view after complete excision and bilateral lymphatic dissection. (**B**) Macroscopic view of the extirpated tumor. (**C**) Macroscopic view of the dissected tumor. (**D**) The 15th postoperative day.

She underwent surgical excision of the recurrent vulvar tumor (complete extirpation of the mons pubis tumor with a 2 cm safety margin) and bilateral inguinal lymphadenectomy (Figure 4A–C). The tumor was solid, lobulated, and yellow-gray on the cut section with a lipomatous appearance (Figure 4C). The overlying skin was not involved, and both the deep and lateral resection margins were free of tumor infiltration. No intraoperative complications; the defect was closed primarily using layered sutures without the use of a flap, wound closure and drainage were completed successfully.

The patient recovered well postoperatively, with no fever, no wound complications, and spontaneous micturition and defecation restored.

Histologically, the morphology of the recurrent tumor closely resembled the primary lesion from 2020, confirming the diagnosis of recurrent high-grade leiomyosarcoma [3]. The recurrent surgical specimen, consisted of a wide local resection encompassing the entire mons pubis and surrounding soft tissues, measuring 150 × 150 mm in total area. Multiple tumor nodules were identified within the subcutaneous tissue, the largest measuring approximately 50–60 mm. On the cut surface, the tumor was solid, lobulated, and yellow-gray in appearance with a fatty texture. There was no involvement of the overlying skin grossly. The deep and lateral margins appeared uninvolved by tumor upon gross examination.

Also included in the resection were bilateral inguinal lymph node packages. The left inguinal region contained a notably enlarged lymph node measuring 25 × 20 mm, while other nodes appeared unremarkable.

**Figure 5:**
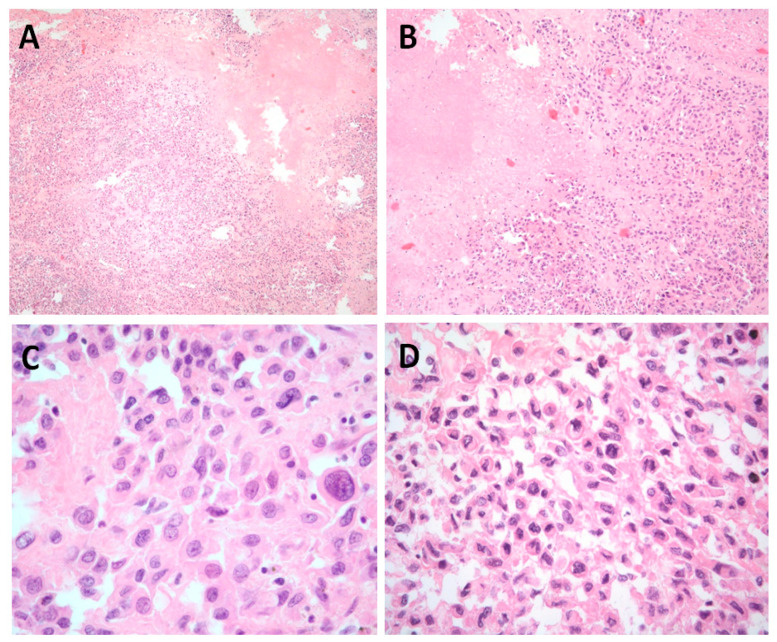
(**A**) HE, 4×—Cellular tumor with evidence of tumor necrosis and cellular pleomorphism. (**B**) HE, 10×—Cellular tumor with evidence of tumor necrosis and cellular pleomorphism, scant lymphocytic infiltrate can be noticed. (**C**) HE 40×—Significant pleomorphism and cellular atypia can be noted. (**D**) HE 40×—Significant pleomorphism and cellular atypia are observed, single mitoses are evident.

Microscopically, the morphology of the recurrent tumor closely resembled the primary leiomyosarcoma excised in 2020, as presented in Figure 5A–D [3]. The tumor consisted of densely cellular, intersecting fascicles composed of spindle-shaped cells with marked nuclear pleomorphism, coarse and irregular chromatin, and prominent nucleoli. The cytoplasm was eosinophilic and moderate in volume, consistent with smooth muscle differentiation. The tumor exhibited high mitotic activity, with numerous mitoses per high-power field, including atypical forms. Scattered areas of coagulative tumor necrosis were present, as well as foci of peritumoral fibrosis. No lymphovascular invasion was identified in the histological specimen.

The resection margins—both lateral and deep—were histologically free of tumor cells. There was no direct extension or presence of tumor infiltration observed in the resection margins. The resection line was at least 20 mm away from the tumor infiltration front. The dermis and epidermis overlying the tumor were not involved, consistent with the absence of skin infiltration observed grossly.

A total of five inguinal lymph nodes were examined. All demonstrated reactive follicular hyperplasia and sinus histiocytosis, with no evidence of metastatic tumor involvement in either the left or right inguinal regions.

Immunohistochemical staining of the recurrent lesion confirmed a consistent profile with the primary tumor from 2020 [3]. In both specimens, tumor cells showed: Smooth Muscle Actin (SMA)–strong, diffuse positivity, confirming smooth muscle differentiation; Vimentin (+) positive, indicating mesenchymal origin; CD34 local positivity; while not specific, this marker is occasionally expressed in soft tissue sarcomas; S100 Protein (-) negative; effectively excluding tumors of neural crest origin such as malignant peripheral nerve sheath tumors or melanoma; MyoD1 (−) negative; ruling out rhabdomyoblastic differentiation. In the 2020 specimen, CD68 demonstrated focal positivity, likely indicative of background histiocytic activity rather than neoplastic expression [3]. This was not a significant feature in the 2025 recurrence.

**Figure 6:**
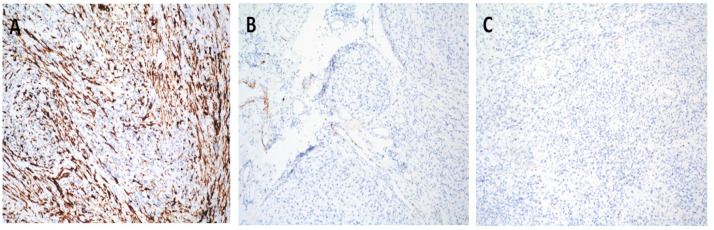
(**A**) Alpha SMA, 100×. (**B**) Desmin, 100×. (**C**) Myo-D1, 100×.

The immunophenotype of the recurrent lesion also demonstrated somewhat partial loss of specific smooth muscle differentiation, with evident but aberrant positivity for alpha-SMA (Figure 6A) and a current lack of Desmin expression (Figure 6B). MyoD1 remained negative (Figure 6C). The current appearance and differentiation closely resemble that of undifferentiated pleomorphic sarcoma.

The immunophenotype remained stable over time, with no significant phenotypic shifts observed between the initial tumor and the recurrence. These findings support the diagnosis of recurrent high-grade leiomyosarcoma with smooth muscle differentiation.

Adjuvant therapy was reinitiated in 2025, but treatment was interrupted after one month due to the development of radiodermatitis and mucoid reactions, limiting the tolerability of further radiotherapy at that time.

## 3. Discussion

**Figure 7:**
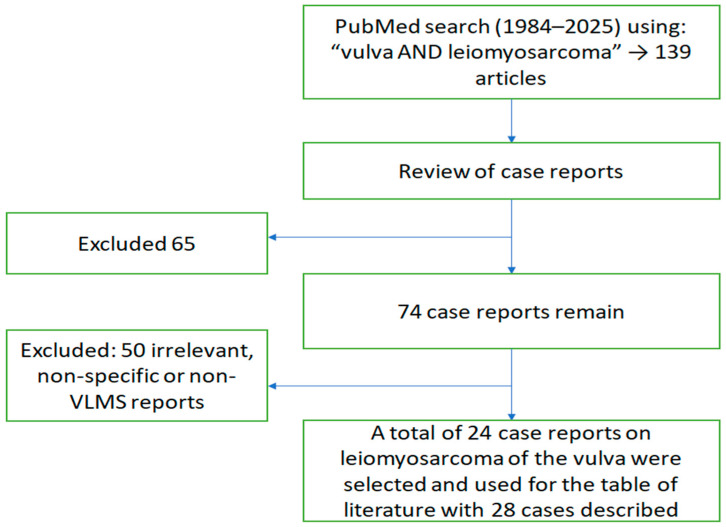
Flowchart of the literature search and selection process for the literature used in the review and discussion.

A literature search was performed to provide an overview of all the cases and case characteristics of VLMS described in the literature. We conducted a search in the PubMed^®^ database from the years 1984 to 2025 for the terms “vulva AND leiomyosarcoma”. This yielded a total of 139 citations. Based on the article type filter-case report, we excluded 65 of them. Of the remaining 74, only 24 meeting the criteria. Thus, a total of 24 publications on LMS of the vulva were taken into account to use for the discussion and review of literature, including the table of literature (Figure 7).

The clinical data and characteristics of the VLMS database are summarized in Table 1 [3,4,5,6,7,8,9,10,11,12,13,14,15,16,17,18,19,20,21,22,23,24,25].

VLMS is a rare malignancy, with only 28 well-documented cases published in the literature between 1980 and 2025. An analysis of these cases reveals several clinical patterns, particularly concerning recurrence risk, tumor behavior, and management outcomes.

Our current case represents a recurrence of the tumor first, described by Yordanov et al. in 2020, five years after the original diagnosis [3]. This recurrence highlights the latent and unpredictable nature of VLMS. While the majority of documented recurrences occur within the first 2–3 years post-treatment, a few cases—including ours—demonstrate that VLMS can recur after prolonged disease-free intervals, underscoring the importance of long-term surveillance.

From the 28 reviewed cases, recurrence was noted in nine patients. Among those, five experienced multiple recurrences, with at least three ultimately succumbing to the disease. Recurrence was often associated with larger tumors (≥8 cm). The mean size of tumors that recurred was approximately 8.5 cm, while non-recurrent tumors averaged closer to 5.2 cm, suggesting a correlation between tumor size and recurrence risk.

Metastasis was less common, reported in 6 out of the 28 cases, typically involving the lungs. Interestingly, even large tumors did not universally metastasize, suggesting variable biological behavior. Notably, the current case presented a substantial recurrence (15 × 10 cm), yet without nodal or distant metastasis, aligning with prior reports that VLMS tends to recur locally before systemic spread.

Treatment strategies varied widely. Most patients underwent surgical excision, ranging from simple tumorectomy (TE) to radical vulvectomy with bilateral lymph node dissection (BILND). Unfortunately, most authors have described their surgical interventions without specifying the safety margins, which are crucial to assess given the rarity of the disease and their importance in predicting patient outcomes. Adjuvant radiotherapy and chemotherapy were employed inconsistently, with no consensus on effectiveness. Cases with radical excision appeared to have better outcomes. In our case, both the primary and recurrent tumors were managed surgically with macroscopically complete resection and histologically negative margins.

Histologically, the recurrent tumor mirrored the initial lesion, demonstrating consistent morphology and immunophenotype across the five-year interval. This lack of dedifferentiation suggests a stable tumor biology in this case. The immunophenotype, although somewhat discrepant with the commonly expected for leiomyosarcoma is consistent with the dedifferentiation observed in leiomyosarcoma of the uterine corpus in the context of tumor progression [26]. According to the present case, dedifferentiation during tumor progression can be observed in the context of leiomyosarcoma of the vulva. Such phenotypic stability, though not universal, may correlate with lower metastatic potential.

Demographically, most affected individuals were postmenopausal women aged 45–74. The most common presenting symptoms included a painless or painful vulvar mass, with occasional lymphadenopathy or skin involvement. However, the clinical resemblance to benign lesions often delayed diagnosis.

Overall, this case contributes to the increasing amount of data indicating that, even in cases that were first thought to be cured, VLMS has a significant risk of late recurrence. It also supports the idea that aggressive surgical management can achieve long-term control, especially when combined with vigilant long-term follow-up.

There is not a systematic approach for the management and follow-up of VLMS because of the small number of patients and the variation in presentation and prognosis. Further multicenter collaboration and centralized case registries may improve our understanding of VLMS and help develop evidence-based guidelines for optimal care.

## 4. Conclusions

High-grade VLMS remains a rare and aggressive malignancy with a notable risk for delayed recurrence. This case, representing a five-year recurrence of a previously reported tumor, demonstrates the necessity for extended surveillance even in the absence of early relapse. The consistent histopathologic features over time, absence of metastasis, and successful surgical re-excision suggest that timely detection and multidisciplinary management can still yield favorable outcomes. This case reinforces the importance of individualized follow-up strategies in VLMS and contributes to the sparse long-term data available for this disease.

## Figures and Tables

**Table 1:** Clinical and morphological features of patients diagnosed with VLMS.

№	Author/Year	Age	Location	Symptoms	Initial Tumor Size (cm)	Recurrence Size (cm)	Primary Treatment	Recurrence Treatment	Recurrence (Yes/No)	Follow-Up (months)	Metastasis
1	Audet-Lapointe et al., 1980 [4]	48	Left labium majus	Painless growing nodule	2 × 4	7 × 2 → 3	TE	TE, Radical Vulvectomy + BILND	Yes (2×)	24	No
2	Smith et al., 1984 [5]	49	n.a.	n.a.	7	n.a.	WLE + RT	Not specified	Yes	18	No
3	KragMoller et al., 1990 [6]	54	Left labium majus	Growing tender swelling	8 × 7 × 4	-	TE, Radical Vulvectomy + BILND	-	No	30	No
4	Kuller et al., 1990 [7]	17	Hymeneal ring, left	No	4 × 4 × 4	-	TE, WLE, BLINK	-	No	20	No
5	Bakri et al., 1992 Case 1 [8]	29	Right labium majus	No	9	Multiple	WLE	4 × WLE	Yes (4×)	120	Lung
6	Bakri et al., 1992 Case 2 [8]	41	Left labium majus	Painless growing nodule	10 × 8	Multiple	WLE	3 × WLE	Yes (3×)	90	Lung
7	Bakri et al., 1992 Case 3 [8]	17	Left labium majus	Lump	10 × 15	Multiple	WLE	2 × WLE	Yes (2×)	108- death	Lung
8	Bakri et al., 1992 Case 4 [8]	20	Left vulva/ groin	Pain/swelling	8 × 10; 5 × 3 (ILN)	n.a.	WLE, Laparotomy	n.a.	n.a.	n.a.	Internal iliac nodes
9	Patel et al., 1993 [9]	n.a.	n.a.	n.a.	n.a.	n.a.	TE	Radical Vulvectomy, BLINK, WLE	Yes (1×)	n.a.	No
10	Dhar et al., 1994 [10]	45	Left labium minus	Itching, pain	4 × 6 × 3	-	FNA ILN, Simple Vulvectomy	-	No	12	No
11	Tawfik et al., 1994 [11]	52	Right labia majora/minora	Pain, swelling	12 × 15	-	TE	-	No	14	No
12	Lösch et al., 2001 [12]	38	Vulva	Slowly growing mass	n.a.	-	TE	-	No	24	No
13	Rawal et al., 2005 [13]	81	Right vulva	Lichen sclerosus over 30 yr, 1 yr postmenopausal, bleeding	5×2	-	WLE	-	No	9	No
14	Androutsopoulos et al., 2005 [14]	55	Right labia majora	5 yr enlargement	8×9	n.a.	n.a.	n.a.	n.a.	6 (Death)	Multiple
15	Dewdney et al., 2005 [15]	36	Vulva	Slowly growing painless mass	n.a.	-	Modified Radical Vulvectomy	-	No	13	No
16	Shankar et al., 2006 [16]	58	Right vulva	Enlarging asymptomatic lump	3	-	TE	-	No	42	No
17	Gonzalez-Bugatto et al., 2009 [17]	52	Left Bartholin gland area	Painless nodule rapid growth	6	3–4	Excision → Hemivulvectomy+ ILND → Adjuvant RT + Chemo	WLE	Yes	48	No
18	McKenzie et al., 2011 [18]	45	Right labium majus	n.a.	n.a.	-	TE	-	No	24	No
19	Levy et al., 2014 Case 1 [19]	50	Left labia inBartholin area	No	4×6	n.a.	TE	-	n.a.	n.a.	n.a.
20	Levy et al., 2014 Case 2 [19]	57	Bartholin area	No	4×2	n.a.	TE	-	n.a.	n.a.	n.a.
21	Alnafisah et al., 2016 [20]	37	Left vulva	Rapid enlarging mass	5.0 × 3.9 × 2.9	Lung	WLE → Radical Vulvectomy + ILND → Chemo → Brachytherapy + EBRT	Lung lobectomy + Chemo	Yes	24+	Lung
22	Korkmaz et al., 2016 [2]	65	Left vulvar Bartholin area	Enlarging vulvar mass	5 × 6	-	TE	-	No	6	No
23	Saquib et al., 2020 [21]	63	Left labia majora	Painless swelling	2.8 × 2.4× 1.4	-	TE	-	No	6	No
24	Yordanov et al., 2020 [3]/Present Case 2025	73/78	Symphysis	Pain, rapid growth	7 × 5	15×10	WLE	Re-excision + LND	Yes	60	No
25	Aljehani et al., 2021 [22]	38 (pregnant)	Left labia majora/minora	Vulvar mass	15 × 10	n.a.	TE	-	No	12	No
26	Reinicke et al., 2022 [23]	14	Left labia majora	Growing mass	6 × 6	n.a.	TE	n.a.	n.a.	n.a.	n.a.
27	Capalbo et al., 2022 [24]	74	Vulva	n.a.	12.5	n.a.	Neoadjuvant Chemo → Radical Vulvectomy → Chemo	-	No	12	No
28	Rathore et al., 2023 [25]	47	Left lower of the vulva	Vulvar swelling	8 × 8	n.a.	Radical Vulvectomy	-	No	8	No

Abbreviations: **FU** = follow-up; **TE** = tumorectomy; **WLE** = wide local excision; **BILND** = bilateral inguinal lymph node dissection; **RT** = radiotherapy; **ILND** = inguinal lymph node dissection **n.a.** = not available.

## Data Availability

Data presented in this study are available upon request from the corresponding author due to privacy concerns.
